
JOURNAL INFORMATION
==============================
NLM Title Abbreviation: ChemistryOpen
Journal ID: ChemistryOpen
NLM Journal ID: 101594811
Journal ID: open

ChemistryOpen

EISSN: 2191-1363
Publisher: Wiley

ARTICLE INFORMATION
==============================
PMCID: PMC3646426
PMID: 24551529
DOI: 10.1002/open.201390006
Article version: 1
Subjects: Graphical Abstract

Graphical Abstract: ChemistryOpen 2/2013

Print publication date: 2013 Apr
Electronic publication date: 2013 Apr 23
Volume: 2
Issue: 2
First page: 41
Last page: 44
Copyright: Copyright © 2013 WILEY-VCH Verlag GmbH & Co. KGaA, Weinheim
Copyright year: 2013
License: Re-use of this article is permitted in accordance with the Creative Commons Deed, Attribution 2.5, which does not permit commercial exploitation.
License URL: https://creativecommons.org/licenses/by/2.5/

==============================
ChemistryOPEN
Including Thesis Treasury

02/2012

ChemistryOPEN... is a multidisciplinary, gold-road, open-access, international forum for the publication of Full Papers and Communications from all areas of chemistry and related fields. *ChemistryOpen* also publishes the Thesis Treasury containing summaries of Ph.D. theses and links to the full version via our homepage. Based in Europe, *ChemistryOpen* attracts authors and readers from around the world, as open-access publishing becomes more important in all areas of chemistry. *ChemistryOpen* is co-owned by ChemPubSoc Europe and published by Wiley-VCH. Authors can submit their primary research articles and thesis summaries via our homepage by clicking "submit an article". All contributions considered suitable for publication are subject to peer review, and if accepted, electronically processed and published online ensuring high quality and short publication times.

Discover this journal online at:
WILEY ONLINE LIBRARY
wileyonlinelibrary.com

Most of the articles in this issue have already appeared online on wileyonlinelibrary.com.
See http://www.chemistryopen.org under EarlyView®

### COVER PICTURE

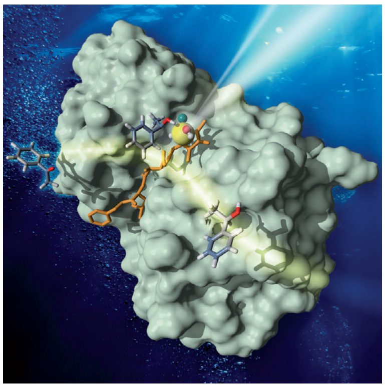

**The cover picture illustrates** the concept of using metal-conjugated affinity labels (m-ALs) to convert proteases into well-defined and catalytically active artificial metalloenzymes. The X-ray structure of the papain-bound inhibitor E64c (orange) served as the basis to predict the orientation of the half-sandwich rhodium(III) moiety (yellow sphere) within the binding pocket of the protease. The well-defined position of the affinity label on the protein surface leads to a distinct environment of the metal center, which translates into enantiomeric ratios of up to 82:18 in the aqueous hydrogenation of ketones. For more details, see the Communication by Jörg Eppinger et al., on p. 50 ff.

WILEY ONLINE LIBRARY © 2013 Wiley-VCH Verlag GmbH & Co. KGaA, Weinheim

ChemistryOPEN Masthead

ChemPubSoc Europe www.chemistryopen.org

Austria
Belgium
Czech Republic
France
Germany
Greece
Hungary
Italy
Netherlands
Poland
Portugal
Slovakia
Spain
Sweden
Switzerland

### Editorial Advisory Board

### *Chairmen*

**Ramón Martínez-Máñez (Spain)**
**Thomas Wirth (UK)**

### *Honorary Chairman*

**Jean-Marie Lehn (France)**

### *Members*

Fernando Albericio (Spain)
Valentin Ananikov (Russia)
Didier Astruc (France)
Jochen Autschbach (USA)
Martin Banwell (Australia)
Vincenzo Barone (Italy)
F. Matthias Bickelhaupt (The Netherlands)
Kathrin Breuker (Austria)
Young Keun Chung (South Korea)
Richard G. Compton (UK)
Ulf Diederichsen (Germany)
Lothar Dunsch (Germany)
Antony Fairbanks (New Zealand)
Ernest Giralt (Spain)
Stefan Grimme (Germany)
Dirk Guldi (Germany)
Akira Harada (Japan)
Narayan Hosmane (USA)
Ken Houk (USA)
Osamu Ito (Japan)
Christoph Janiak (Germany)
Kazunori Kataoka (Japan)
Yoshiaki Kiso (Japan)
Mats Larhed (Sweden)
Lechoslav Latos-Grażyński (Poland)
Thisbe Lindhorst (Germany)
Luis M. Liz-Marzán (Spain)
Carlos Lodeiro (Portugal)
Todd L. Lowary (Canada)
Jean Martinez (France)
Pedro Matias (Portugal)
Stefan Matile (Switzerland)
Pedro Molina (Spain)
K. C. Nicolaou (USA)
Berit Olofsson (Sweden)
Oliver Reiser (Germany)
Kari Rissanen (Finland)
Knut Rurack (Germany)
James Rusling (USA)
Albert Schenning (The Netherlands)
Robert Schlögl (Germany)
Patrick Schmuki (Germany)
Ulrich S. Schubert (Germany)
Min Shi (China)
Norio Shibata (Japan)
Rint Sijbesma (The Netherlands)
Choong Eui Song (South Korea)
Petr Štěpnička (Czech Republic)
Brian Stoltz (USA)
Steve Suib (USA)
Reshef Tenne (Israel)
Matthew Todd (Australia)
Claudio Toniolo (Italy)
Jaume Veciana (Spain)
Hans-Achim Wagenknecht (Germany)
Ralph Weissleder (USA)
Gunther Wittstock (Germany)
Vivian W. W. Yam (China)
Chun-Hua Yan (China)
Jincai Zhao (China)

### Open Access and Copyright

All research articles published by *ChemistryOpen* are fully open access: immediately freely available to read, download and share. All *ChemistryOpen* articles are published under the terms of the Creative Commons Attribution Non-Commercial License which permits use, distribution and reproduction in any medium, provided the original work is properly cited and is not used for commercial purposes.

Copyright on any research article published by *ChemistryOpen* is retained by the author(s). Authors grant Wiley a license to publish the article and identify itself as the original publisher. Authors also grant any third party the right to use the article freely as long as its integrity is maintained and its original authors, citation details and publisher are identified.

### Use by commercial "for-profit" organisations

Use of Wiley Open Access articles for commercial, promotional, or marketing purposes requires further explicit permission from Wiley and will be subject to a fee. Copyright 2012 Wiley-VCH Verlag GmbH & Co. KGaA, Weinheim. All rights reserved. Requests to reproduce material from this journal content should be addressed to rights@wiley-vch.de, and reprints of *ChemistryOpen* articles can be purchased from chem-reprints@wiley-vch.de.

### Disclaimer

The Publisher and Editors cannot be held responsible for errors or any consequences arising from the use of information contained in this journal; the views and opinions expressed do not necessarily reflect those of the Publisher and Editors, neither does the publication of advertisements constitute any endorsement by the Publisher and Editors of the products advertised.

Wiley Open Access articles posted to repositories or websites are without warranty from Wiley of any kind, either express or implied, including, but not limited to, warranties of merchantability, fitness for a particular purpose, or non-infringement. To the fullest extent permitted by law Wiley disclaims all liability for any loss or damage arising out of, or in connection with, the use of or inability to use the content.

**Editors:**
Karen Hindson, Haymo Ross

**Deputy Editor:**
Natalia Ortúzar

**Associate Editor:**
Saskia Neubacher

**Secretary:**
Vanessa Müller

**Production:**
Stefanie Klein, Margitta Schmitt

**Telephone:**
(+ 49) 6201-606-305

**Fax:**
(+ 49) 6201-606-203

**E-mail:**
chemistryopen@wiley-vch.de

**Courier Services:**
Boschstrasse 12
69469 Weinheim (Germany)

**Regular Mail:**
Postfach 10 11 61
69451 Weinheim (Germany)

**Homepage:**
http://www.chemistryopen.org

**Manuscript Submission & Personal Homepage**

manuscriptXpress

**http://www.manuscriptxpress.com**

**Customer Services:**
www.wileycustomerhelp.com

**Marketing:**
Monika Silz (ext. 458, fax: ext. 100,
e-mail: msilz@wiley-vch.de)

**Advertising:**
Marion Schulz (ext. 565, fax: ext. 550,
e-mail: mschulz@wiley-vch.de, jspiess@wiley-vch.de)

**Copyright Permissions:**
Bettina Loycke (ext. 280, fax: ext. 447,
e-mail: rights@wiley-vch.de)

**Reprints, e-prints, posters:**
Carmen Leitner (fax: ext. 226)
e-mail: chem-reprints@wiley-vch.de

**Publishers:**
WILEY-VCH Verlag GmbH & Co. KGaA,
Postfach 10 11 61
69451 Weinheim (Germany)
http://www.wiley-vch.de/

**Publication:**
6 issues in 2013

© 2013 WILEY-VCH Verlag GmbH & Co. KGaA, Weinheim.

### COVER PROFILE

*"We reasoned that covalent approaches so far suffered from an ill-defined orientation of the metal center on the protein surface. Using specifically designed metal-conjugated affinity labels to introduce metal centers into the binding pocket of cysteine proteases, we generate a structurally distinct binding mode of the cofactor."*
This and more about the story behind the cover research can be found on p. 50.

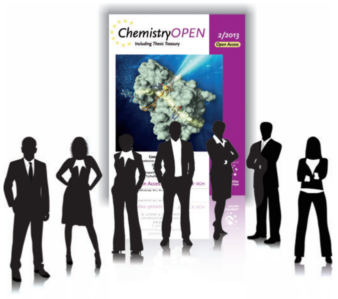

*T. Reiner, D. Jantke, A. N. Marziale, A. Raba, J. Eppinger**

***40***

**Metal-Conjugated Affinity Labels: A New Concept to Create Enantioselective Artificial Metalloenzymes**

### NEWS

Spotlights on our sister journals ***46 – 49***

### COMMUNICATIONS

**How to train a protein:** Metal-conjugated affinity labels were used to selectively position catalytically active metal centers in the binding pocket of proteases. The resulting artificial metalloenzymes achieve up to 82 % e.r. in the hydrogenation of ketones. The modular setup enables a rapid generation of artificial metalloenzyme libraries, which can be adapted to a broad range of catalytic conditions.

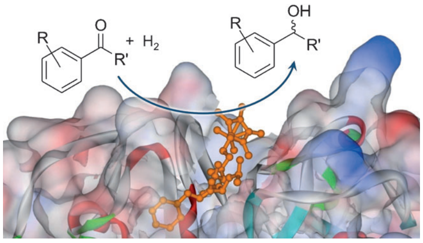

*T. Reiner, D. Jantke, A. N. Marziale, A. Raba, J. Eppinger**

***50 – 54***

**Metal-Conjugated Affinity Labels: A New Concept to Create Enantioselective Artificial Metalloenzymes**

**Like beads on a string:** A synthetic method for the directional construction of strings of spherical fullerenes along stacks of planar oligothiophenes is described. The key to success was the preparation of fullerenes with two solubilizing tri(ethylene glycol) tails (bold) and an aromatic aldehyde for covalent capture by hydrazides along the oligothiophene stacks (red).

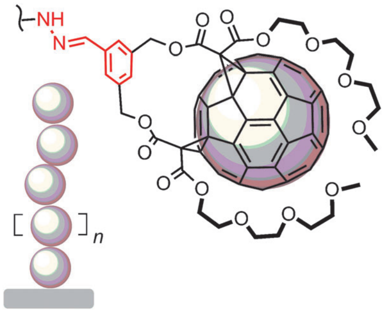

*A. Bolag, H. Hayashi, P. Charbonnaz, N. Sakai, S. Matile**

***55 – 57***

**Self-Organizing Surface-Initiated Polymerization of Multicomponent Photosystems: Stack Exchange with Fullerenes**

© 2013 Wiley-VCH Verlag GmbH & Co. KGaA, Weinheim

*S. Elsayed, A. Agostini, L. E. Santos-Figueroa, R. Martínez-Máñez,* F. Sancenón*

*58 – 62*

**An Instantaneous and Highly Selective Chromofluorogenic Chemodosimeter for Fluoride Anion Detection in Pure Water**

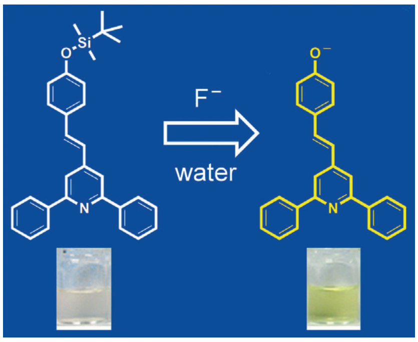

**The fast and the fluoride:** A pyridine derivative functionalized with *tert*-butyldimethylsilyl ether has been synthesized and used as a selective chromofluorogenic fluoride sensor in water/cetyltrimethylammonium bromide solutions. The chromofluorogenic response arises from the fluoride-induced hydrolysis of the silyl ether moiety, which generates a coloured and highly emissive phenolate anion.

### FULL PAPERS

*W. Yuan, Y. Wei, M. Shi**

*63 – 68*

**Ruthenium-Catalyzed Intramolecular [2+2+2] Cycloaddition and Tandem Cross-Metathesis of Triynes and Enediynes**

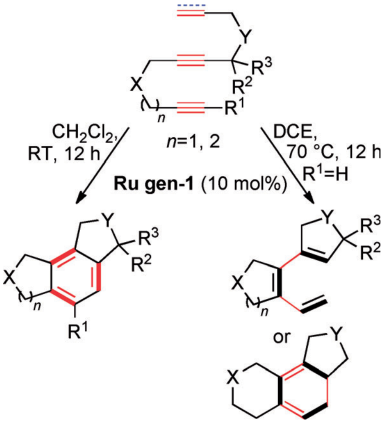

**Back to generation one!** Catalyzed by the first-generation Grubbs ruthenium complex, intramolecular [2+2+2] cycloaddition of triynes to their benzene derivatives **2** and tandem cross-metathesis reactions of enediynes to their conjugated triene derivatives **4** perform smoothly in moderate to good yields under mild conditions. A possible reaction mechanism is presented on the basis of deuterium-labeling results.

*J. Ellison, K. Tschulik,* E. J. E. Stuart, K. Jurkschat, D. Omanović, M. Uhlemann, A. Crossley, R. G. Compton**

*69 – 75*

**Get More Out of Your Data: A New Approach to Agglomeration and Aggregation Studies Using Nanoparticle Impact Experiments**

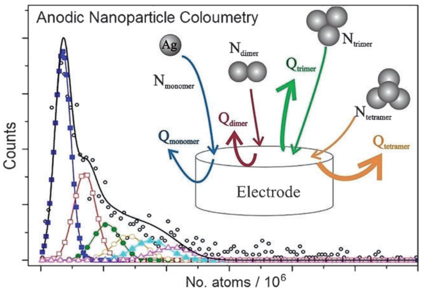

**The impact of Ag nanoparticles:** A new approach to both sizing nanoparticles in solution and determining their agglomeration and aggregation state solely by electrochemical methods is presented. The data obtained by this method is critically compared to results of nanoparticle tracking analysis.

* Author to whom correspondence should be addressed.

Supporting information on the WWW (see article for access details).

This is an open-access article, published under the terms and conditions of the Creative Commons Attribution Non Commercial License.

A video clip is available as Supporting Information on the WWW (see article for access details).

VIP Contributions labeled with this symbol have been judged as "Very Important Papers" by the referees.

### SERVICE

**All the Tables of Contents may be found on the WWW under: http://www.chemistryopen.org**

© 2013 Wiley-VCH Verlag GmbH & Co. KGaA, Weinheim
